# Challenges and Perspectives of Chemical Biology, a Successful Multidisciplinary Field of Natural Sciences

**DOI:** 10.3390/molecules16032672

**Published:** 2011-03-23

**Authors:** Fernando A. Rojas-Ruiz, Leonor Y. Vargas-Méndez, Vladimir V. Kouznetsov

**Affiliations:** 1 Laboratorio de Química Orgánica y Biomolecular, Facultad de Ciencias, Universidad Industrial de Santander, Bucaramanga, Colombia; E-Mail: fernandorojas799@gmail.com; 2 Grupo de Investigaciones Ambientales, Facultad de Química Ambiental, Universidad Santo Tomás, A. A. 1076, Bucaramanga, Colombia; E-Mail: leyavar@gmail.com

**Keywords:** chemical biology, small molecule library, chemical sensibilization

## Abstract

Objects, goals, and main methods as well as perspectives of chemical biology are discussed. This review is focused on the fundamental aspects of this emerging field of life sciences: chemical space, the small molecule library and chemical sensibilization (small molecule microassays).

## 1. Introduction

**Figure 1:**
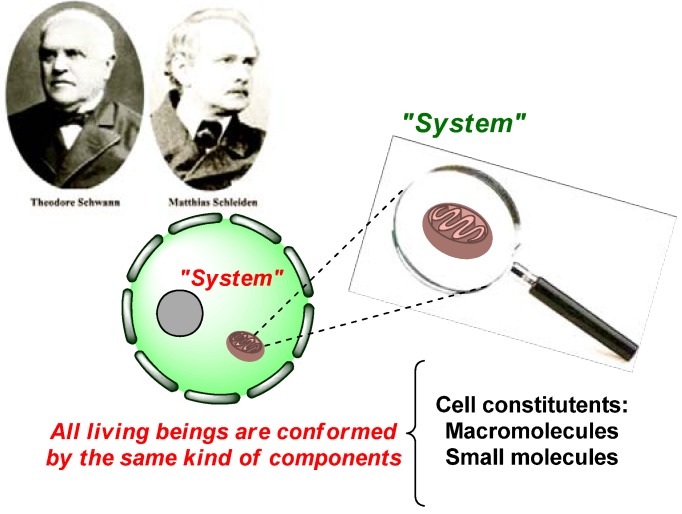
Cellular objects of chemical biology.

Chemistry, biology and physics are the essential pillars sustainingthe development of the natural sciences. The enormous progress in our understanding of biological systems, currently in expansion, is due to the skillful application of the principles and techniques of organic chemistry, wherebysynthetic organic chemistry plays the initiator role in the biological discovery. This shows that biology “has moved” from the descriptive (phenomenological) level to the molecular (biochemistry) level generating new disciplines (structural biology, molecular biology) that now form part of the field of natural sciences. Living organisms produce and releasechemical compounds into the environment significantly affecting other organisms and determining the existence of chemical interactions between these individuals. That is to say, all different organisms generate chemical signals and, in return, every single one responds to some other organism’s chemical signal. Chemical signals produced by organisms are made up of compounds produced through secondary metabolic pathways that are intimately related to primary metabolic pathways and metabolites (carbohydrates, lipids, proteins, and nucleic acids). In biology, the analysis of these interactions might be performed “from up to down” in the direction of decreasing complexity of a biological system. For example, “top-down” analysis begins with a cell, a tissue, a limb, or an organism itself, and ends at the molecular level with the molecules that participate in its complex intra- and/or interactions. From chemistry’s molecules and native macromolecules “bottom-up” synthesis begins in the direction of increasing complexity to reach the totality of the cell and its higher organizations emerging through modular motives and supramodular functional units [1].Since 1839 it has been recognized (Schwann and Schleiden) that the cell is the simplest unit in living organisms. Furthermore, the cell is a protected region, in which diverse small molecules and macromolecular clusters (both kinds of molecules are endogenous) interact with each other in a harmony that is reached by auto-assembling. Much of the cell’s content is an aqueous solution with small molecules (e.g., simple sugars, amino acids, vitamins) and ions (e.g., sodium, chloride, calcium ions) [2]. In this sense, to perform further studies on living systems and biochemical processes, there was a need to have available tools to disrupt these systems using small molecules and, therefore, find a new depth and detailed information on the living systems operation (Figure 1).

Following this trend, a new discipline, chemical biology, appeared from the interface between the synthetic organic chemistry and the molecular, structural, and cellular biology. Chemical biology’s principal task was to explain the fundamental ideas related to life chemistry and to apply the knowledge of living organism’s behaviors to its interactions between biological macromolecules (endogens) and small organic molecules (exogens). This means going beyond the understanding of biological processes to the molecular level.

Chemical biology differs from biochemistry (biological chemistry) principally in its chemical analysis methods of secondary metabolism products and their interconvertions. Chemical biology also differs from bio-organic chemistry, whose action field is the secondary metabolism products study. Chemical biologists are largely using organic chemistry techniques in exploring biological systems and understanding how biological systems work (mechanism, *etc*), whereas biochemists use techniques closer to biology to understand interactions of biomolecules on the descriptive level, generally.

**Figure 2:**
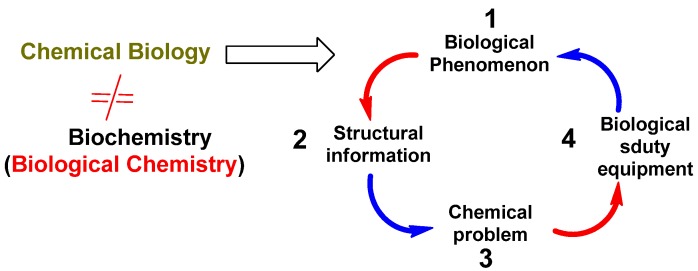
Principal stages of the study of chemical biology.

The initial stage of chemical biology consists on the analysis of a biological system or phenomenon of interest (Figure 2). In this analysis structural information concerning the structure of biomolecules involved in a particular biological phenomenon, or the structure of endogenous small molecules which interact with these macromoleculesare deduced, for instance. Without structure, identifying the function of the system is complicated. This structural information is then employed to define unsolved chemical problems, i.e. the development of new methods for the synthesis of small molecules like secondary metabolites or inhibitors that can be used to perturb and examine biological systems. Without its synthesis there will not be enough material to study the structure and the process dynamic neither. The last stage consists in the use of the prepared molecules as instruments within adequately designed biological or biochemical experiments [3,4] (Figure 2).

The objects, objectives, principal methods, and perspectives of chemical biology are discussed in this review. Emphasis is placed on the central aspects of this emerging field of life science which include the chemical space, small molecule library, and chemical sensibilization (small moleculemicroarrays) [5], trying to make it more illustrative. This review does not pretend to be complete; giving the chemical biology bases and advances in the limited space of this journal format is almost unattainable. The principal goal of the present revision is to encourage young organic chemistry researchers’ interest through the synthetic organic chemistry – biology interface and to demonstrate the multidisciplinary research importance of this new area that has been gaining momentum worldwide.

## 2. Chemical Space and Biological Space

The expression “small molecules” appeared during the development of organic compound synthetic methodologywithreference to their molecular weight (less than 500–700 Da). Synthetic or natural molecules are used as pharmacological prototypes (models) or as precursors in the construction of new chemical entities of wide and diverse practical utility. They also can be used as crucial instruments to study biological processes.

The virtual chemical world of small molecules as well as natural macromolecules is immense; therefore studying them is an arduous task [6,7,8,9]. However, dynamic interactions of organic chemistry and biology have led to identify certain molecular structures that are widely employed within the “natural laboratory” repertory. Besides being important in the studies of small molecules and natural macromolecular associations, these molecular structures are known as “privileged structures” [10]. The term “privileged structure” was introduced by Evans in 1988, and it is defined as “a molecular structure able to provide different receptor ligands” [11].

Accordingly, synthetic small molecules (synthetically derived by chemists) or natural small molecules (metabolite products of organisms) with cell membrane permeability capacity can be used to modulate protein functions in a selective, rapid and reversible way.

Molecules are characterized by a wide rangeof descriptors, such as shape, physical properties (molecular mass, nucleophilicity, lipophilicity, and dipolar moment), topology, *etc* [12,13,14]. In this sense, the term “chemical space” is equivalent to the “multi-dimensional descriptor space” that enshrines all of the carbon small molecules, which in principle, could be created. This means that within a chemical space there are structural or molecular characteristics that determine an organic compound’s family [5].

**Figure 3:**
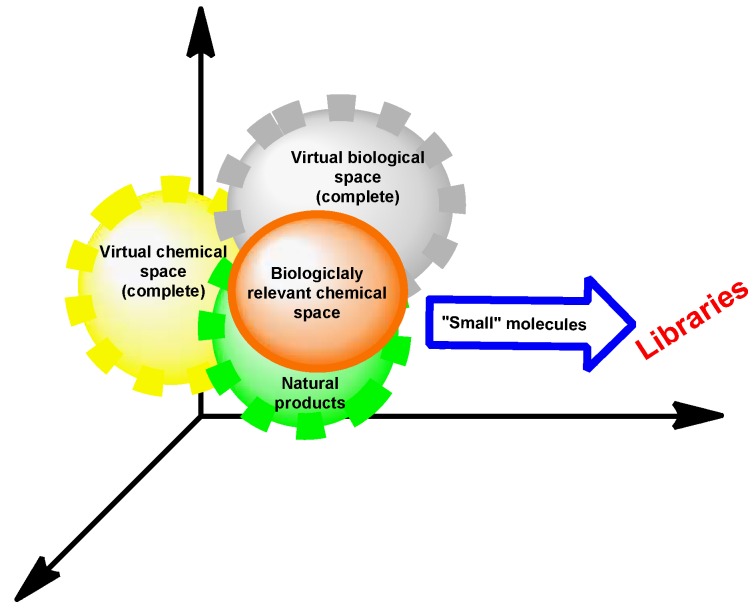
Chemical and biological space relationship.

On the other hand, it is calculated that the virtual library of chemical compounds with pharmacological properties could be approximately 10^60^ bioactive molecules [15,16], although the chemical compounds used by biological systems represent only a very small fraction of this astronomic number and they have small molecular mass. It is assumed that the simplest living organisms can auto-organize with some hundred different types of these compounds; while the most complex organisms must contain thousands of different small molecules [5]. Thus, it is clear in terms of the number of compounds, that the biologically relevant chemical space is a very small fraction of complete chemical space that may contain 10^30^–10^200^ possible small molecules [17,18] according to the calculated parameters (Figure 3).

At the same time, it is important to recognize that nowadays there are approximately 49,000,000 substances registered by the Chemical AbstractsService (CAS) [19] and only 1,350 pharmaceuticals based on the small molecules approved by the U.S. FDA [20]. Living systems have evolved over a billion years to materialize carefully the controlled chemistry in an aqueous media typically at temperatures between 0–100 °C. Under these conditions that are essential for life, many chemical reactions do not occur with an appreciable rate and most of them would not yield the products in a reproducible and specific way. Therefore, these chemical reactions require an additional and vital component, called an enzyme. Enzymes, together with other proteins and diverse nucleic acids are used by the living systems to undergo the realization and control of these reactions. These macromolecules are responsible for the synthesis, transport, and degradation of every small molecule within the biological environment. Now it is known that the genomes of the simplest living systems encode the sequences of less than 1,000 different proteins, while humans and all mammals have around 50,000 genes, this means that as a rough order of magnitude, an estimated of 50,000 to 100,000 active proteins exist in mammalian bodies, numbers that are a small fraction when compared with the total number of proteins that could theoretically exist. For example, the average size of a natural typical protein is about 300 residues (α-amino acids). If only the 20 canonical α-amino acids come together in various combinations to produce proteins, the number of possible α-amino acid combinations in this 300 amino acids protein model is 20 raised to 300 (20^300^) or 10^390^, and if only a single molecule of each of these polypeptides were to be produced, their combined mass would vastly exceed that of the known Universe. Natural proteins are therefore also a very select group of molecules [5] (Figure 3).

The emergence of macromolecules, which possess the ability to store, distribute information, and translate it into a catalytic function, manifests the dual multi-faceted nature of protein synthesis: as a chain of enzymatic steps of the chemical pathway in the biochemical space and as a process of genetic information transfer in the space of molecular biology.

Being in the biologically relevant chemical space, natural compounds, or natural product-like small molecules play an important role as simple instruments to understand intracellular signaling and protein-protein or protein-DNA dynamic interaction processes, which are common and fundamental to any normal cellular process and to cellular deregulation process. Secondary and primary metabolites co-evolved together– proteins and nucleic acids –and its molecular scaffolds and functional groups “were adjusted” during millions of years for a specific biochemical purpose. For this reason, natural products and their synthetic analogues encompass this biologically relevant chemical space and have high affinities to their respective biological targets.

## 3. Small Molecules Library Generation

There are three sources that allow obtaining small molecules that could form libraries: (1)isolation of natural products, (2)chemical or/and chemo-enzymatic derivation of natural products, and (3)chemical synthesis [21,22]. Traditionally, natural products are usually studied as a complex extract mixture that is subjected to rigorous separation processes, analysis, and spectroscopic study, in addition to evaluation of their biological properties. This process conduces to the identification of lead molecules that can act as pharmacologic agents, because natural products are indisputable models for chemical synthesis and chemical biology.

Chemical synthesis (preparation of new molecules by means of chemical reactions) has been and still is an important procedure for the generation of new molecular libraries. Chemical synthesis possesses several strategies and tactics that develop gradually based on the demand of other sciences.

**Figure 4:**
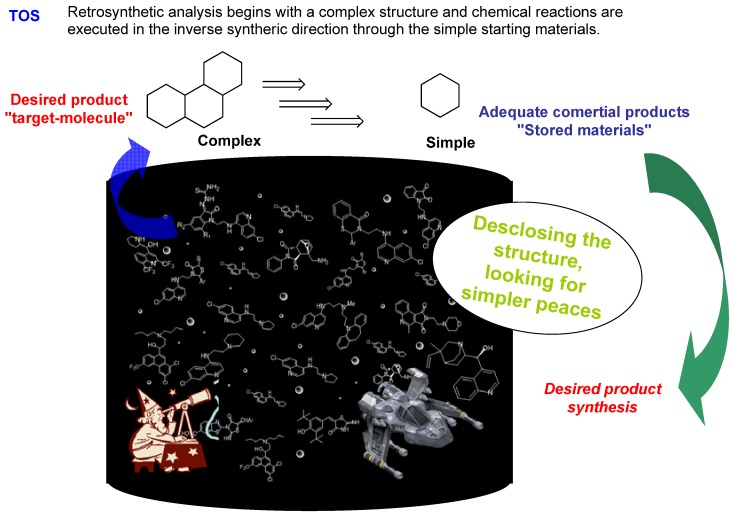
TOS methodology.

The first strategy, known as synthesis oriented towards a specific target (or desired product) - Target Oriented Synthesis (TOS), allows the access to a specific region of chemical space. This approach is intimately linked to the retro-synthetic analysis development [23], which begins with the disconnection of a complex structure looking for some simple and appropriated materials to reach the preparation of the structurally complex molecule (Figure 4).

The retro-synthetic analysis of a complex product allows preparing it via a disconnection process where this product is "broken" down into chemical species that can be synthesized from available substrates using known reactions. This “top-down retro-synthesis” process is opposite to chemical synthesis.

As it was mentioned above, synthetic organic chemistry explores a dense region of chemical space in a precise area with known properties. However, are these chemical space regions defined by a natural product or a known structure, really the best or most fertile region for the discovery of small structures able to modulate macromolecular functions? This is a highly relevant question for organic chemists, taking into account the high potentiality offered by the small molecules.

**Figure 5:**
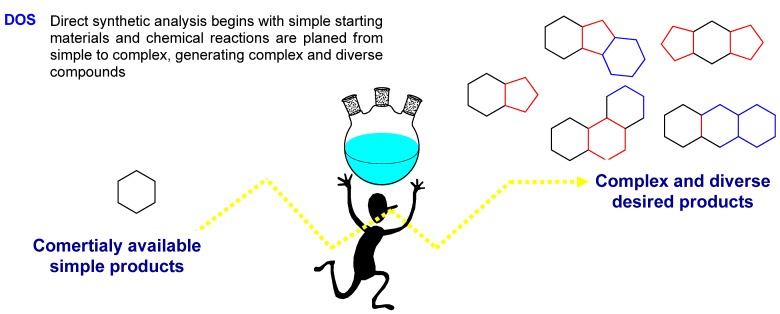
DOS methodology.

The answer is found inside the principles of the synthetic tendency, known as Diversity Oriented Synthesis (DOS), that allows a wide compound distribution within the chemical space [24,25]. This methodology provides deliberate, simultaneous, and efficient synthesis of more than one target-compound in a diversity-directed approach to respond to a complex problem [26] and allows the construction of small molecule collections showing a range of bioactivities pointing to the efficient synthesis of molecules with diverse and different molecular structures [27]. Although the structural complexity is not a requirement for molecular diversity, it has been proposed to confer specificity within biological interactions [28]. In the DOS methodology, the synthetic analysis is performed “forward” and the strategy is developed so the simple row materials can be transformed into diversified and complex compounds [29] (Figure 5).

Members of the DOS library should be “diverse” in their own substitutions, as well as in the location of these substitutions. In this sense, to design a DOS methodology, it is necessary to bear in mind the four types of diversification [30,31]:

- Substituent diversity: this can be incorporated by a “combinatorial variation” within the employed building blocks.
- Stereochemical diversity: this can be incorporated using agents able to control the asymmetric reactions.
- Functional group diversity: this can be included, by chemical manipulation.
- Nuclei diversity: this allows the different ring’s fusion and formation.

The DOS methodology includes the use of sequential reactions capable of generating complexity and incorporating molecular diversity inside a compound’s collection to form simple starting materials [32]. As a result, in these “branching pathways”, the product of one reaction is the substrate for the following step, transforming a simple row material in a diverse and complex molecular series. The total synthesis of complex natural products (TOS) as well as the structurally diverse libraries (DOS) requires strategies and tactics with well-defined characteristics. These synthetic methods must be robust, flexible, and stereoselectives [33].

The increasing interest in the synthesis of heterocyclic molecule libraries obligates chemists to develop new strategies for the promissory libraries design that could be generated employing different models. An important question must be done before selecting such a model: how many molecules must a collection contain to be productive and structurally diverse? There are “large” libraries (with more than one million compounds) [34] and “short” libraries (with only 10,000 compounds) [35], both guided by one natural product, whose principal task is to generate a lead-compound, a pharmacological agent, in an effective, rapid, and economic way. However, a common misconception is that the most large and diverse collections are automatically better; moreover in practical terms, “large” libraries (10^10^ molecules) are difficult to organize based on the properties of each compound. It seems that it could be suitable to generate and examine a “small or short” collection (less than 60 compounds) of alkaloid-like molecules or terpenoids. A “structurally diverse” ideal library containing 40 natural diverse molecules has been theoretically evaluated and shown to have superior parameters compared to collections with 46–168 members [36].

To go deeper into biological processes at a molecular level, new libraries focused on Biology Oriented Synthesis (BIOS) are needed as well [37]. The main requirement for these types of libraries, based on the natural product bioactive structures, is to study the biological systems by means of direct perturbations using small molecules [38,39]. The depicted small molecule advantages - high temporal control, good and easy dosage control, and versatility allow for the measurement of biological responses rapidly in a wide variable range for different cell species (systems) *in vivo* and *in vitro*.

In summary, it is necessary to highlight the vital importance of natural products and/or its close analogues in revealing biological mechanisms, furthermore emphasize that almost all of them are contained by the biologically relevant chemical space (Figure 3). Natural products have great affinity towards macromolecules, *e.g.*, proteins, DNA, and lipid structures, products of primary metabolism. At the same time, various classes of compounds, forexample, terpenes, phenolics, phenylpropanoids or alkaloids, playa prominent role in secondary metabolism. Therefore, one of these promissory products could be an initial prototype in the generation of its analogues via BIOS methodology [40,41,42,43]. Thus, the new natural products inspired libraries are efficient and promising in the pharmacologically active agents research. Within the scientific literature more than 50 libraries based on natural product frameworks are found [43,44]. Some of these molecular collections are based on the combinatorial chemistry idea [45,46,47].

## 4. Chemical Sensibilization

Since one of the prime targets of chemical biology is to exploit the power of synthetic organic chemistry to discover and explain the essential molecular pathways in cellular, molecular, and structural biology, modern preparation methods are needed for new small molecules that will be the main instruments in these studies. 

**Figure 6:**
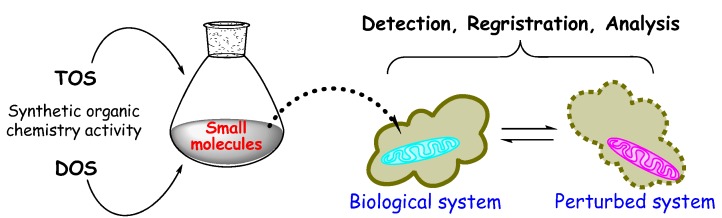
A simplified scheme of chemical biology study.

Having these instruments, new micro bioassay techniques are needed (chemical sensibilization), which will be able to detect new changes through functional perturbation using small molecules and to answer biological questions (Figure 6).

Foremost, different measure formats can be employed to explore these perturbations in a highly rational and efficient way [48]. Within the development of a small molecules screening test, three critical factors must be considered: (i) test type (biochemical, cellular, phenotypic, micro assay *etc*.); (ii) detection technology (luminescence, fluorescence, radioactive,*etc*.), and (iii) required reagents to be employed (cell lines, enzymatic substrates, purified proteins, antibodies, and positive or/and negative controls, *etc*.). There are several formats, diverse shapes and sizes; nevertheless, these can be broadly classified in three different categories: (a) High-Throughput Screens (HTS); (b) High-Content Screens (HCS) and c) Small-Molecule Microarrays (SMM). The first process, where numerous molecules are analyzed in a swift and parallel way to uncover bioactivity, was developed thanks to combinatorial chemistry [49,50]. The second process is based on the cell or organism analysis by image automatized techniques to detect multiple phenotypic responses. The latter is more attractive and trendy today [51,52]. In these sorts of assessments, generally, small molecules are covalently linked to the micro assay surface (glass, gel, polymer, *etc*.) and exposed to the target of interest. These assessments allow for the identification of novel modulators for different proteins within several biological processes. 

**Figure 7:**
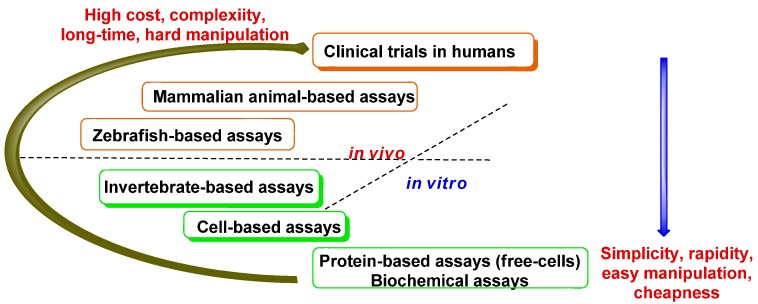
A diagram ofthe types of bioassays.

In the “physiological context”, these assays are now automated and work well for cell free systems (enzymes, proteins, DNA *in vitro*), nonetheless the *in vivo* assays based on vertebrate mammalian cellular tissues are very difficult and expensive long-time process (Figure 7). 

The intermediate level between enzymatic assays and assays on the cellular tissues of mammalian vertebrates could be an invertebrate model assay (*Caenorhabditiselegans*, *Drosophila melanogaster* and *Daniorerio*) [53], because they are relative simple processesinvolving easy manipulations. However, in comparison to two first models, the zebrafish model for small-molecule discovery is more similar to mammalian orthologs. The zebrafish [54,55,56,57] instance can be classified as a “border line instance” (Figure 7). This last method is productive especially over the developmental biology [58], fields such as chemical genetics [59] and oncology [60,61], among others.

One of the SMM methods and thereby, of chemical biology, is the design and preparation of small molecules, whosemolecular mechanism (mode of action) is based on the inactivation of enzymes implicated in diverse diseases, including parasitic, infectious, *etc* [62]. During biochemical research, it is well-known that any small molecule that slows down or blocks enzyme catalysis (reversiblyor irreversibly) is an enzyme inhibitor. These molecules must be structurally similar to the substrate for a specific enzyme. If the interaction with the target enzyme is irreversible (usually covalent), then the small molecule is referred to as an enzyme inactivator (or irreversible inhibitor). Many natural products and/or theirclose analogues work as enzyme inhibitor or inactivators. As the name implies, inhibition of an enzyme activity by a reversible inhibitor is reversible, suggesting that noncovalent interactions are involved. An irreversible inhibitor (enzyme inactivator) can prevent the return of the enzymatic activity for an extended period of time, suggesting the involvement of a covalent bond [62]. Among all the target proteins for potential therapeutic use, enzymes are the most promising for rational inhibitor design. Thus, the discovery of new selective enzyme inhibitors is an exciting approach to the rational discovery of new drugs [62,63,64]. These molecules can be designed using an organic-synthetic rational approach founded on natural products.

In general, most of these compounds perform as base models (prototypes) for pharmaceutical development [65,66] and are invaluable precursors in cellular biology [67]. Moreover, by use of small molecules similar to natural products as protein-protein interaction modulators it can be understood more about the complex intracellular signaling processes.

To ensure an effective small molecule library designed by BIOS methodology, there are three synthetic strategies: (1) molecular scaffold based libraries of a particular natural product (alkaloid, phytohormone, *etc*.); (2) libraries derived from natural product sets with specific substructures; (3) libraries determined by the natural product’s structural characteristics resemblance. The three strategies provide positive results and interesting examples [37,68].

**Figure 8:**
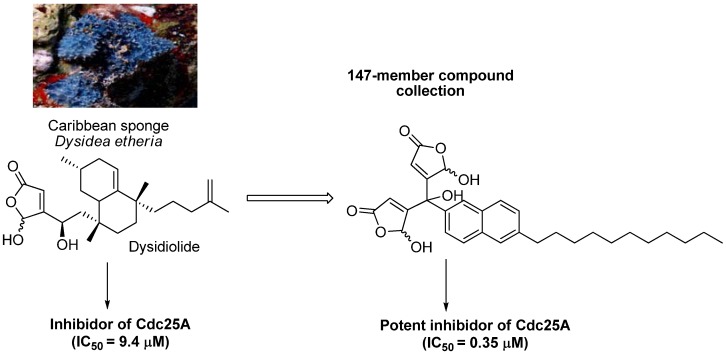
Development of a new potent inhibitor from anatural product.

Most BIOS/SSM research is committed to the new natural product analogues or chemotherapeutic agents’ discovery with improved and concrete properties. Waldmann and Schreiber´ studies illustrate this working chart. Prof. Waldmann and co-workers have shown a small molecule collection based on the sesquiterpenedysidiolide structural framework isolated from the Caribbean sponge *Dysideaehteria* exhibiting an inhibitory activity against a phosphatase protein Cdc25A. During this study a compound with 27 times more activity was found, retainingthe γ-hydroxybutenolide structural moiety [69] (Figure 8).

**Figure 9:**
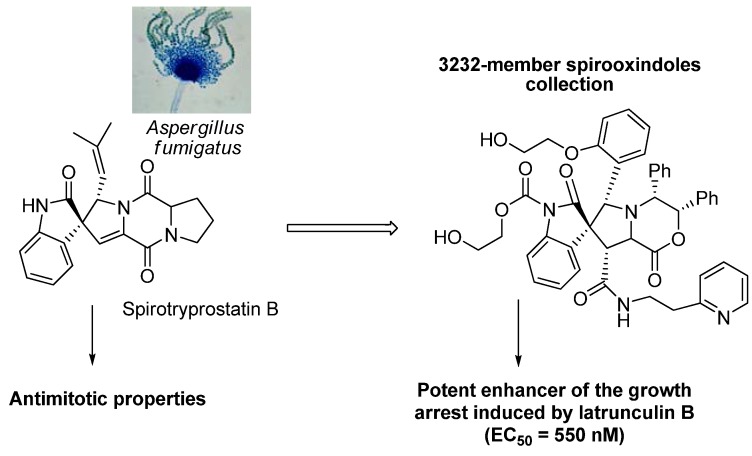
New spirooxindole collection inspired by the spirotryprostatin B structure.

The research group directed by Prof. Schreiber took an indole alkaloid spirotryprostatin B as a prototype in a new library study of 3,232 spirooxindole molecules. This molecule, isolated from the saprophyte mold *Aspergilliusfumigatus*, has been found to have antimitotic properties. By developing yeast assay it was possible to identify enhancers of growth arrest induced by latrunculin B (a natural product that sequesters monomeric actin and prevents the formation of actin microfilaments), new spirooxindols with enhancer properties were synthesized [70] (Figure 9).

**Figure 10:**
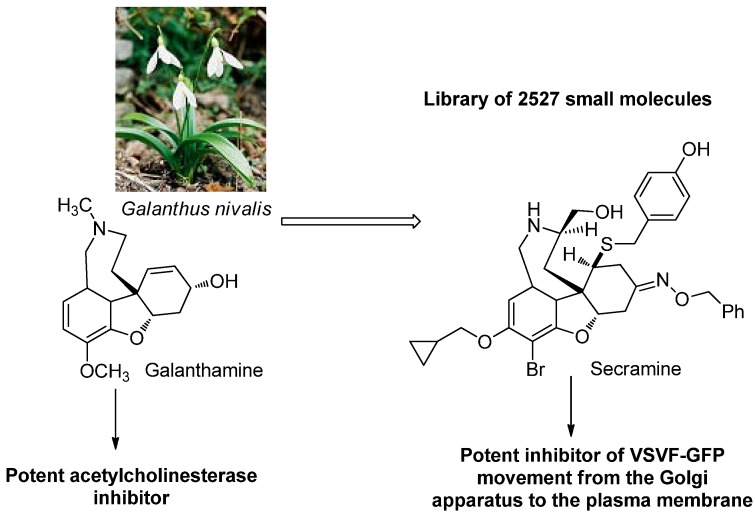
Development and discovery of VSVF-GFP inhibitor via BIOS methodology.

In the effortsof Shair and co-workers [71], a small (2,527) library of molecules was developed inspired by the alkaloidgalantamine, a potent AChE inhibitor. It is interesting that the alkaloid structure was selected because of its high range of functionality and molecular rigidity, and not because of its potent activity, while looking for molecules proficient in perturbing the protein traffic from the endoplasmic reticulum to the plasma membrane through the Golgi apparatus. Employing a phenotypic cellular assay with SMM screening, it was possible to identify new molecule, secramine that is a potent VSVG-GFP movement inhibitor from the Golgi apparatus to the plasma membrane (Figure 10).

Within the experiment designed by Schreiber and co-workers [72], a SMM of 12,396 molecules library was used, based on specific substructures from complex natural products, evaluation with the fusion protein Hap3p-GTS resulted in the discovery of the cellular transcription inhibitor named haptamide B. A further study by Schreiber and co-workers, focused on the new bioactive molecules identification with a 1,3-dioxane sub-structure effecting phenotypic tests and several enzymatic bioassays (50 different biotests) [73]. Using a similar strategy the Schlutz group prepared a new *N*-heterocyclic library containing 45,140 distinct molecules with the purine sub-structure [74]. Recently, a SMM method was reported within the A549 and HeLa mammalian cell-based screening format with an imaging-based readout. This method will be a valuable support on the discovery of new potential chemotherapeutic agent [75].The above mentioned and selected examples are just a few of the vast studies number of such developed by scientists working in the chemical biology field.

## 5. Future

Chemical biology must demonstrate how reactions and small molecules will be able to be used in a fascinating way to study biology. In general it must also show how it analyzes the structures and functions of materials produced by chemical or biological means. Chemical biology, a synthetic or modified small molecule science, within the live systems context is now able to go beyond the memory and cognition, the detection and signaling, and the comprehension and modulation studies of cellular circuits. Without a doubt chemical biology is now capable of and will achieve success in the discovery ofnew biological phenomena, expanding our knowledge horizons about living beings, including ourselves [76].

## References

[1] Quinkert G., Wallmeier H., Windhab N., Reichert D., Schreiber S.L., Kupoor T.M., Günther W. (2007). Chemical Biology From Small Molecules to Systems Biology and Drug Design.

[2] Lodish H., Berk A., Kaiser C.A., Krieger M., Scott M.P., Bretscher A., Ploegh H., Matsudaira P. (2008). Molecular Cell Biology.

[3] Waldmann H., Janning P. (2004). Chemical Biology, A Practical Course.

[4] Sekhon B.S. (2008). Chemical Biology: Past, Present and Future. Curr. Chem. Biol..

[5] Gordon E.J. (2007). Small-Molecule Screening: It Takes a Village. ACS Chem. Biol..

[6] Dobson C. (2004). Chemical space and biology. Nature.

[7] Haggarty S.J. (2005). The principle of complementary: Chemical versus biological space. Curr. Opin. Chem. Biol..

[8] Wetzel S., Klein K., Renner S., Rauh D., Oprea T., Mutzel P., Waldmann H. (2009). Interactive exploration of chemical space with Scaffold Hunter. Nat. Chem. Biol..

[9] Renner S., Otterlo W., Dominguez M., Möcklinghoff S., Hofmann B., Wetzel S., Schuffenhauer A., Ertl P., Oprea T., Steinhilber D. (2009). Bioactivity-guided mapping and navigation of chemical space. Nat. Chem. Biol..

[10] Horton D.A., Bourne G.T., Mark L., Smythe M.L. (2003). The Combinatorial Synthesis of Bicyclic Privileged Structures or Privileged Substructures. Chem. Rev..

[11] Evans B.E., Rittle K.E., Bock M.G., DiPardo R.M., Freidinger R.M., Whitter W.L., Lundell G.F., Veber D.F., Anderson P.S., Chang R.S.L., Lotti V.J., Cerino D.J., Chen T.B., Kling P.J., Kunkel K.A., Springer J.P., Hirshfield J. (1988). Methods for drug discovery: Development of potent, selective, orally effective cholecystokinin antagonists. J. Med. Chem..

[12] Pitt W.R., Parry D.M., Perry B.G., Groom C.R. (2009). Heteroaromatic Rings of the Future. J. Med. Chem..

[13] Rush T.S., Grant J.A., Mosyak L., Nicholls A. (2005). A Shape-Based 3-D Scaffold Hopping Method and Its Application to a Bacterial Protein-Protein Interaction. J. Med. Chem..

[14] Rosén J., Lovgren A., Kogej T., Muresan S., Gottfries J., Backlund A. (2009). ChemGPS-NPWeb: Chemical space navigation online. J.Comput-Aided Mol. Des..

[15] Kirkpatrick P., Ellis C. (2004). Chemical space. Nature.

[16] Bohacek R.S., McMartin C., Guida W.C. (1996). The art and practice of structure-based drug design: A molecular modeling perspective. Med. Res. Rev..

[17] Van Deursen R., Reymond J.L. (2007). ChemicalSpaceTravel. Chem. Med. Chem..

[18] Fink T., Reymond J.L. (2007). Virtual Exploration of the Chemical Universe up to 11 Atoms of C, N, O, F: Assembly of 26.4 Million Structures (110.9 Million Stereoisomers) and Analysis for New Ring Systems, Stereochemistry, Physicochemical Properties, Compound Classes, and Drug Discovery. J. Chem. Inf. Model.

[19] CAS A Division of the American Chemical Society. http://www.cas.org/cgi-bin/cas/regreport.pl..

[20] U.S. FDA U.S. Department of Health and Human Services. http://www.fda.gov/Drugs/DrugSafety/PostmarketDrugSafetyInformationforPatientsandProviders/UCM111085..

[21] Clardy J., Walsh C. (2004). Lessons from Natural Molecules. Nature.

[22] Schreiber S.L. (1998). Chemical genetics resulting from a passion for synthetic organic chemistry. Bioorg. Med. Chem..

[23] Corey E.J., Cheng X.M. (1995). The Logic of Chemical Synthesis.

[24] Schreiber S.L. (2000). Target-Oriented and Diversity-Oriented Organic Synthesis in Drug Discovery. Science.

[25] Burke M., Schreiber S.L. (2004). A planning strategy for Diversity-Oriented Synthesis. Angew. Chem. Int. Ed..

[26] Spring D. (2003). Diversity-oriented synthesis, a challenge for synthetic chemists. Org. Biomol. Chem..

[27] Spadl R., Spring D., Bender A. (2008). Diversity-oriented synthesis; a spectrum of approaches and results. Org. Biomol. Chem..

[28] Lipinsky C., Hopkins A. (2004). Navigating chemical space for biology and medicine. Nature.

[29] Spandl R., Díaz-Gavilán M., O’Connell K., Thomas G., Spring D. (2008). Diversity-oriented synthesis. Chem. Rec..

[30] Burke M., Gerger E., Schreiber S.L. (2004). A synthesis strategy yielding skeletally diverse small molecules combinatorially. J. Am. Chem. Soc..

[31] Burke M., Berger E., Schreiber S.L. (2003). Generating diverse skeletons of small molecules combinatorially. Science.

[32] Thomas G., Spandl R., Glansdorp F., Welch M., Bender A., Cockfield J., Lindsey J., Bryant C., Brown D., Loiseleur L., Rudyk H., Ladlow M., Spring D. (2008). Anti-MRSA agent discovery using Diversity-Oriented Synthesis. Angew. Chem. Int. Ed..

[33] Shaw T. (2009). Naturally diverse: Highlights in versatile synthetic methods enabling target- and diversity-oriented synthesis. Nat. Prod. Rep..

[34] Tan D.S., Foley M.A., Shair M.D., Schreiber S.L. (1998). Stereoselective synthesis of over two million compounds having structural features both reminiscent of natural products and compatible with miniaturized cell-based assays. J. Am. Chem. Soc..

[35] Nicolaou K.C., Pfefferkorn J.A., Mitchell H.J., Roecker A.J., Barluenga S., Cao G.Q., Affleck R.L., Lillig J.E. (2000). Natural product-like combinatorial libraries based on privileged structures.2. Construction of a 10 000-membred benzopyran library by directed split-and-pool chemistry using NanoKans and optical encoding. J. Am. Chem. Soc..

[36] Fergus S., Bender A., Spring D.R. (2005). Assessment of structural diversity in combinatorial synthesis. Curr. Opin. Chem. Biol..

[37] Tan D.S. (2005). Diversity-oriented synthesis: Exploring the intersections between chemistry and biology. Nat. Chem. Biol..

[38] Stockwell B. (2004). Exploring biology with small organic molecules. Nature.

[39] Shang S., Tan D.S. (2005). Advancing chemistry and biology through diversity-oriented synthesis of natural product-like libraries. Curr. Opin. Chem. Biol..

[40] Aray P., Joseph R., Gan Z., Rakic B. (2005). Exploring New Chemical Space by Stereocontrolled Diversity-Oriented Synthesis. Chem. Biol..

[41] Quinn R.J., Carroll A.R., Pham N.B., Baron P., Palframan M.E., Suraweera L., Pierens G.K., Muresan S. (2008). Developing a Drug-like Natural Product Library. J. Nat. Prod..

[42] Kumar K., Waldmann H. (2009). Synthesis of Natural Product Inspired Compound Collections. Angew. Chem. Int. Ed..

[43] Boldi A.M. (2004). Libraries from natural product-like scaffolds. Curr. Opin. Chem. Biol..

[44] Nandy J.P., Prakesch M., Khadem S., Reddy P.T., Sharma U., Arya P. (2009). Advances in Solution- and Solid-Phase Synthesis toward the Generation of Natural Product-like Libraries. Chem. Rev..

[45] Gordon E.M., Barrett R.W., Dower W.J., Fodor S.P.A., Mark A., Gallop M.A. (1994). Applications of Combinatorial Technologies to Drug Discovery. 2. Combinatorial Organic Synthesis, Library Screening Strategies, and Future Directions. J. Med. Chem..

[46] Armstrong R.W., Combs A.P., Tempest P.A., Brown S.D., Keating T.A. (1996). Multiple-Component Condensation Strategies for Combinatorial Library Synthesis. Acc. Chem. Res..

[47] Zhang W., Luo Z., Chen C.H.-T., Curran D.P. (2002). Solution-Phase Preparation of a 560-Compound Library of Individual Pure Mappicine Analogues by Fluorous Mixture Synthesis. J. Am. Chem. Soc..

[48] Liu B., Li S., Hu J. (2004). Technological advances in high-throughput screening. Am. J. Pharmacogenomics.

[49] Ortholand J.-Y., Ganesan A. (2004). Natural products and combinatorial chemistry: Back to the future. Curr. Opin. Chem. Biol..

[50] Rademann J., Jung G. (2000). Intergrating Combinatorial Synthesis and Bioassays. Science.

[51] Uttamchandani M., Walsh D.P., Yao S.Q., Chang Y.T. (2005). Small molecule microarrays: Recent advances and applications. Curr. Opin. Chem. Biol..

[52] Chiosis G., Brodsky J.L. (2005). Small molecule microarrays: From proteins to mammalian cells – are we there yet?. Trends Biotechnol..

[53] Ségalat L. (2007). Invertebrate Animal Models of Diseases as Screening Tools in Drug Discovery. ACS Chem. Biol..

[54] Lovel D.R., Pichler F.B., Dodd A., Copp B.R., Greenwood D.R. (2004). Technology for high-throughput screens: The present and future using zebrafish. Curr. Opin. Biotechnol..

[55] Sumanas S., Lin S. (2004). Zebrafish as a model system for drug target screening and validation. Drug Discov. Today Targets.

[56] Nicholson R.L., Welch M., Ladlow M., Spring D.R. (2007). Small-Molecule Screening: Advances in Microarraying and Cell-Imaging Technologies. ACS Chem. Biol..

[57] MacRae C.A., Peterson R.T. (2003). Zebrafish-Based Small Molecule Discovery. Chem. Biol..

[58] Peterson R.T., Link B.A., Dowling J.E., Schreiber S.L. (2000). Small molecule developmental screens reveal the logic and timing of vertebrate development. Proc. Natl. Acad. Sci.USA.

[59] Ahn Y.H., Chang Y.T. (2007). Tagged Small Molecule Library Approach for Facilitated Chemical Genetics. Acc. Chem. Res..

[60] Goessling W., North T.E., Zon L.I. (2007). New Waves of Discovery: Modeling Cancer in Zebrafish. J. Clin. Oncol..

[61] Feitsma H., Cuppen E. (2008). Zebrafish as a Cancer Model. Mol. Cancer. Res..

[62] Silverman R.B. (1992). The Organic Chemistry of Drug Design and Drug Action.

[63] vonAhsen O., Bomer U. (2005). High-throughput screening for kinase assay. ChemBioChem.

[64] Kumar R.A., Clark D.S. (2006). High-throughput screening of biocatalytic activity: Application in drug discovery. Curr. Opin. Chem. Biol..

[65] Newman D.J., Cragg G.M., Snader K.M. (2000). The influence of natural products upon drug discovery. Nat. Prod. Rep..

[66] Newman D.J., Cragg G.M. (2007). Natural Products as Sources of New Drug over the Last 25 Years. J. Nat. Prod..

[67] Schreiber S.L. (1992). The small-molecule approach to biology. Chem. Eng. News.

[68] Reayi A., Arya P. (2005). Natural product-like chemical space: Search for chemical dissectors of macromolecular interactions. Curr. Opin. Chem. Biol..

[69] Koch M.A., Wittenberg L.O., Basu S., Jeyaraj D.A., Gourzoulidou E., Reinecke K., Odermatt A., Waldmann H. (2004). Compound library development guided by protein structure similarity clustering and natural product structure. Proc. Natl. Acad. Sci.USA.

[70] Lo M.M.C., Neumann C.S., Nagayama S., Peristein E.O., Schreiber S.L. (2004). A Library of Spirooxindoles Based on a Stereoselective Three-Component Coupling Reaction. J. Am. Chem. Soc..

[71] Pelish H.E., Westwood N.J., Feng Y., Kirchhausen T., Shair M.D. (2001). Use of Biomimetic Diversity-Oriented Synthesis to Discover Galanthamine-Like Molecules with Biological Properties beyond Those of the Natural Product. J. Am. Chem. Soc..

[72] Koehler A.N., Shamji A.F., Schreiber S.L. (2003). Discovery of an Inhibitor of a Transcription Factor Using Small Molecule Microarrays and Diversity-Oriented Synthesis. J. Am. Chem. Soc..

[73] Sternson S.M., Louca J.B., Wong J.C., Schreiber S.L. (2001). Split-Pool Synthesis of 1,3-Dioxanes Leading to Arrayed Stock Solutions of Single Compounds Sufficient for Multiple Phenotypic and Protein-Binding Assays. J. Am. Chem. Soc..

[74] Ding S., Gray N.S., Wu X., Ding Q., Schultz P.G. (2002). A Combinatorial Scaffold Approach toward Kinase-Directed Heterocycle Libraries. J. Am. Chem. Soc..

[75] Baley S.N., Sabatini D.M., Stockwell B.R. (2004). Microarrays of small molecules embedded in biodegradable polymers for use in mammalian cell-based screens. Proc. Natl. Acad. Sci.USA.

[76] Marcaurellle L.A., Foley M.A. (2010). The evolving role of molecular diversity in drug discovery. Curr. Opin. Chem. Biol..

